# Psychological Well-Being in Nursing Students: A Multicentric, Cross-Sectional Study

**DOI:** 10.3390/ijerph18063020

**Published:** 2021-03-15

**Authors:** Sílvia Reverté-Villarroya, Laura Ortega, Laia Raigal-Aran, Esther Sauras-Colón, Roser Ricomà-Muntané, David Ballester-Ferrando, Carolina Rascón-Hernán, Teresa Botigué, Ana Lavedán, Luis González-Osorio, Ximena Osorio-Spuler, Maria Dolors Burjalés-Martí

**Affiliations:** 1 Departament d’Infermeria, Facultat d’Infermeria, Universitat Rovira i Virgili, Avinguda Remolins, 13-15, 43500 Tortosa, Spain; silvia.reverte@urv.cat; 2 Unitat d’Estudis Clínics, Institut d’Investigació Sanitària Pere Virgili, Hospital de Tortosa Verge de la Cinta, Carretera Esplanetes, 14, 43500 Tortosa, Spain; esther.sauras@iispv.cat; 3 Departament d’Infermeria, Facultat d’Infermeria, Universitat Rovira i Virgili, Avinguda Catalunya, 35, 43002 Tarragona, Spain; laia.raigal@urv.cat (L.R.-A.); roser.ricoma@urv.cat (R.R.-M.); mdolors.burjales@urv.cat (M.D.B.-M.); 4 Hospital Universitari Institut Pere Mata, IISPV, CIBERSAM, Carretera de l’Institut Pere Mata, 43206 Reus, Spain; 5 Faculty of Nursing, University of Girona, Emili Grahit, 77, 17071 Girona, Spain; david.ballester@udg.edu (D.B.-F.); carolina.rascon@udg.edu (C.R.-H.); 6 Nursing and Physiotherapy Department, University of Lleida, 2 Montserrat Roig St., 25198 Lleida, Spain; teresa.botigue@udl.cat (T.B.); ana.lavedan@udl.cat (A.L.); 7 Health Care Research Group (GRECS), Biomedical Research Institute of Lleida, 80 Alcalde Rovira Roure St., 25198 Lleida, Spain; 8 Departamento de Medicina Interna, Oficina de Educación en Ciencias de la Salud, Facultad de Medicina, Universidad de La Frontera, Manuel Montt 112, Temuco 4811230, Chile; luis.gonzalez@ufrontera.cl; 9 Departamento de Enfermería, Facultad de Medicina, Universidad de La Frontera, Manuel Montt 112, Temuco 4811230, Chile; ximena.osorio@ufrontera.cl

**Keywords:** anxiety, depression, mental health, problem-based learning, stress psychological, students, nursing

## Abstract

In addition to complying with strict academic standards, nursing students must acquire relevant knowledge and skills, and learn how to carry themselves in different and often stressful professional settings. These obligations could severely affect their mental health. The purpose of this study was to examine the mental health status of undergraduate nursing students and related factors. A total of 1368 nursing students from different universities in Spain and Chile were included in this study, which took place over the 2018–2019 academic year. We assessed their levels of stress related to specific learning methodologies and determined their mental health status using the General Health Questionnaire (GHQ-28). The results revealed that the more advanced the course was, the lower the total GHQ-28 score. The stress generated by different types of training activities had a significant effect on the total GHQ-28 score. These results suggest that nursing education could act as a protective factor against mental health disorders. Although a heavy academic workload could lead to higher levels of stress, overall, it seems that mental health is better in more advanced courses than in initial academic years.

## 1. Introduction

It has been shown that health professionals experience high levels of stress and depression, related to the high pressure and the handling of complex situations, and a high prevalence of psychiatric comorbidities, such as anxiety or depression [1,2]. In nursing students, we find a doubly risky situation as they are both health professionals and students [3]. It has been estimated that between 21% and 43% of undergraduate students suffer from depression [4]. A meta-analysis conducted by Tung [1] showed high rates of depression in nursing students, with Asia being the location with the highest percentages (43%), followed by Europe (38%), and, in last place, Latin America (21%).

University education involves changes in a student’s lifestyle and conditions, which could lead to stress [5]. Stress is one of the main factors that can trigger discomfort in students and has been related to poorer psychological well-being; social support, self-efficacy, resilience, and mindfulness have a positive effect on mental health [6,7,8]. It has also been found that being employed acts as a protective factor in students enrolled in nursing education programs [9]. Furthermore, there are negative factors that predict psychological problems in students, such as a family history of mental illness, low socioeconomic status, and living far from their families [4]. It is known that stressful situations may directly affect physical and mental health. The symptoms associated with stress are usually accompanied by adjustment, anxiety, and behavioral, sleeping, and emotional disorders [10]. In addition, a lack of sleep affects memory and decreases alertness, and if it becomes chronic it can lead to disorders such as anxiety and depression, and even vascular comorbidity [11].

The academic workload, especially clinical practice, has been identified as the main source of stress in nursing students, followed by financial and family issues [12,13]. Furthermore, professional nursing training includes a wide array of learning methods, which combine clinical practice with putting the competencies acquired into practice [14]. According to the results obtained by McCarthy et al. in their study of academic stress, they identified these main stress factors: the academic environment, exams, activities and projects, and, more specifically, meeting deadlines and the number of exams required. Additionally, students’ concern about their performance in their exams, the large number of students in the classrooms, boring lectures, and feeling doubted by professors were all identified as sources of stress [13]. Suicidal ideation and lower psychological well-being are also associated with academic stress [15].

Adequate psychological well-being is important to completing training [7]; and, identifying psychosocial factors—along with other sociodemographic and academic—that could affect their health, depending on the academic year, could prove very beneficial. Some studies have shown that one of the factors associated with stress is the academic year [12,16,17]). The association between stress and psychiatric morbidities has been widely studied with tools such as the General Health Questionnaire-28 (GHQ-28), which provides a simple method of collecting information on different health problems that could allow us to study the potential effects of stress on psychological well-being [18].

**Figure 1:**
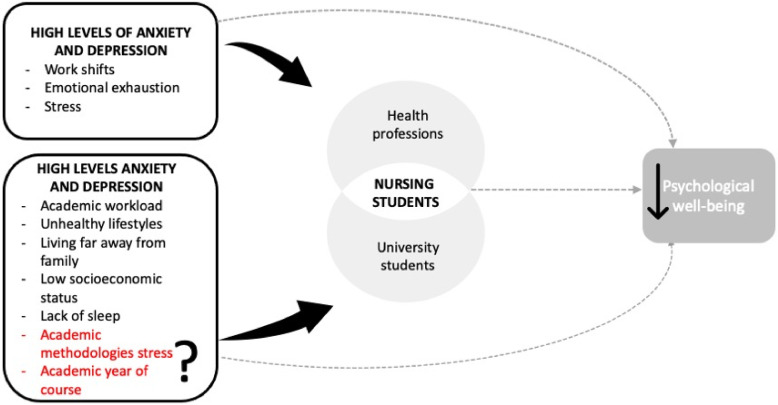
Conceptual framework of the study. On the left, in black, are the factors that have already been shown to directly affect mental health in both health professionals and college students. In red are the specific academic conditions that can affect psychological health depending on the academic year, but which remain uncertain.

Studying the academic stress factors possibly associated with mental health disorders could provide us with valuable information to try to mitigate strain in students in different years throughout the nursing education curricula. The conceptual framework of the study is represented in Figure 1. This could be the first step, academically, to promoting mental health while identifying those students suffering from mental health disorders. Therefore, quick diagnosis and treatment can be provided, consequently preventing a further decline in their mental health or poor educational performance. Our study fills a gap in the literature by comparing students’ well-being in different years of the nursing degree in a multicentric sample and considering specific academic factors related to stress, like different learning methodologies and sociodemographic data—for example, age, gender, university access, employment status or academic funding.

The objectives of this study were to examine the degree of psychological well-being in nursing students in different courses of the nursing degree, to study the association between the stress caused by the different academic methodologies, and to assess the psychological well-being of students.

## 2. Materials and Methods

### 2.1. Design

This multicenter study used a descriptive and correlational cross-sectional design. It is part of a larger-scale longitudinal study carried out in Spain and Chile.

We used a STrengthening the Reporting of OBservational studies in Epidemiology (STROBE) checklist (see Table S1).

### 2.2. Setting and Participants

A total of 1358 undergraduate students from three nursing schools in Spain (University of Lleida-UdL, University RoviraVirgili-URV, University of Girona-UdG) and one from Chile (University of La Frontera-UFRO) participated in this study. The researchers who conducted this study are university professors and have direct access to the different courses of the nursing degree. All undergraduate students were offered the chance to voluntarily participate in the study and those recruited were selected through convenience sampling. The eligibility criteria included all ages, consent to participate in this study, understanding the objectives of the study, and the full ability to communicate verbally in Spanish (the language used in the four universities). The data were collected between March and April of 2018 (academic year 2017–2018). The datasets obtained and analyzed in this study are not publicly available but can be provided upon reasonable request.

### 2.3. Variables and Data Measurements

We collected the sociodemographic and academic characteristics of the students and asked them about the stress caused by different learning methodologies.

The level of stress was categorized as follows: (1) None; (2) Low; (3) Moderate; and (4) High. The types of methodologies included in this study were: lectures, seminars, problem-based learning (PBL), simulation laboratories, clinical practices, group projects, exams, written work, and oral presentations.

We used the General Health Questionnaire (GHQ-28) [19] in the Spanish version [20], one of the most widely used screening instruments in the world to assess self-perceived mental well-being and to detect those individuals who are likely to suffer from or may develop psychiatric disorders. This 28-item questionnaire contains four subscales: (A) Somatic symptoms, (B) Anxiety and Insomnia, (C) Social Dysfunction, and (D) Severe Depression, and each subscale contains seven items. Each item has four possible answers: “much worse than usual”, “worse than usual”, “same as usual”, and “better than usual” [21]. Two different scoring methods were used [21]: the GHQ-28 method (binary) and the Likert scale scoring. The GHQ-28 method (0-0-1-1) scores 0 points in choices 1 and 2, and scores 1 point in choices 3 and 4, with a cutoff of 5–6 by category (the higher the score, the greater the psychological morbidity). In a four-point Likert scale, the possible scores range between 0 and 3, with a minimum score of 0 and a maximum score of 84. The questionnaire correctly identifies 83% of cases with a cutoff of 5–6 (sensitivity 84.6%, specificity 82%), suggesting a discriminatory power almost as good as the other Spanish GHQ versions [20]. The internal consistency of the items included in the GHQ-28 instrument was evaluated through the Cronbach’s alpha coefficient (α = 0.766). Using the last method, we considered a total score of 23–24 as the threshold for the presence of mental health problems [22].

### 2.4. Data Analysis

The sociodemographic and academic characteristics of the participants were analyzed using descriptive statistics. To test differences between the programs, one-way ANOVA or Kruskal–Wallis tests were used for quantitative variables, depending on the normality of the data. A Chi-squared test was used for qualitative variables. Finally, a logistic regression model was conducted to examine which sociodemographic and academic characteristics, together with different stress-inducing methods, resulted in a total GHQ-28 score higher than 23. This logistic regression model was validated using bootstrap techniques. The significance level was established at *p* < 0.05. All data obtained from the questionnaire were analyzed using SPSS version 26.0 (Statistical software, IBM Corp., Armonk, NY, USA).

### 2.5. Ethical Approval and Consent to Participate

The participation of the subjects in the survey was voluntary, and all the responses were anonymized. The questionnaire included an introductory statement regarding the purpose, intent, and use of data.

This study was approved by the Ethics and Biosafety Committee of the University of Girona. All the procedures were performed in accordance with the principles of the 1964 Helsinki Declaration and its later amendments. All participants involved in the study signed written informed consent. The ID protocol of the study was CEBRU0015-2019 code 07/2019.

## 3. Results

The number of participants from each university was: 319 (23.5%) from UdL, 387 (28.5%) from UdG, 491 (36.2%) from URV, and 161 (11.9%) from UFRO. The median age of the nursing students was 21 years (IQR 3.0), and almost 85% of them were between 18 and 24 years old. Differences in age between the academic years were relevant, as age increases with each academic year.

Regarding the access route to tertiary studies and to the participating universities, this study showed that the differences between programs were statistically significant. Therefore, subsequent analyses exploring mental health were carried out separately by each participating university. Other academic and financial variables that presented statistically significant differences were the transfer of academic files, clinical practice experience, working status, and funding systems. Funding received from the family greatly decreased with advancement in the career (Table 1).

The median total GHQ-28 score was 23 (IQR 15.0). We observed statistically significant differences between the first and fourth year, noting that fourth-year students scored lower than first-year students. Median scores for each dimension of the questionnaire (somatic symptoms, anxiety and insomnia, social dysfunction, and depression) were: 5 (IQR 3.0), 6 (IQR 3.0), 6 (IQR 2.0), and 1 (IQR 2.0), respectively. Higher scores were obtained for anxiety, insomnia, and social dysfunction, while lower scores were obtained when assessing depression. Likewise, differences between academic years were statistically significant in terms of social dysfunction (*p* = 0.007) and depression (*p* = 0.001) (Table 2). When analyzing GHQ-28 scores separately by university (data not shown), we found relevant differences in the scores at specific subscales according to the different academic years at the three Spanish universities. UdG obtained statistically significant differences in most subscales: somatic symptoms (*p* < 0.001), anxiety and insomnia (*p* < 0.001), depression (*p* = 0.001), and total GHQ-28 score (*p* < 0.001), where 4th year presented lower scores than 1st year.

Likewise, we gathered information about the level of stress caused by different types of training activities. Higher stress scores were seen when asked about exams and oral presentations, while lower stress scores were obtained in relation to lectures and seminars. In all items, there were statistically significant differences between academic years, except for oral presentations, in which scores remained high without any variation (Table 3).

Finally, the logistic regression model showed that stress generated by seminars, PBL, group projects, and exams had a statistically significant, direct effect on the total GHQ-28 score (Table 4). Gender seemed to impact the GHQ-28 score as well, with men being more likely to score lower than 23 (OR = 0.655, *p* = 0.022). There were no statistically significant differences between the three Spanish universities depending on the academic characteristics. However, UFRO had a probability three times higher of having a GHQ-28 score above 23, compared to UdL (OR = 3.008).

## 4. Discussion

One of the main results of this study was the identification of the sociodemographic and academic characteristics and the learning methodologies that are associated with lower psychological well-being in our students. Regarding prevalence, despite having a small percentage of men in the sample, the results of our study are in line with previous studies, in which men obtained lower GHQ-28 scores than women [24,25].

Furthermore, the more advanced the subjects were in their career, the lower the GHQ-28 score, which decreases the possibility of suffering from psychiatric disorders. It appears that the differences in the GHQ-28 general score obtained in our sample show that final-year students might develop better stress-managing strategies, allowing them to successfully cope with daily life and academic stressors. This could be related to the changes in curricula introduced by the Bologna Process, which took place in Europe during 2010–11 [26], after which new student-based methods were included in school programs throughout Spain. Therefore, it is indisputable that final-year nursing students have wider knowledge of health and lifestyle factors—which may contribute to better psychological well-being—and of the different pathologies caused by stress and inefficient coping mechanisms [27]. Another possible explanation could be derived from the findings of a study in Spain [28] in which researchers observed positive changes in terms of coping strategies, levels of stress, and personality traits in some nursing students during their three-year training program.

Moreover, despite Spain being one of the countries with the lowest nurse-to-patient ratio [29], the employment opportunities of recent nursing graduates is significantly higher [30,31]. Self-confidence upon graduating from university, the prospect of performing fewer tasks simultaneously, and the possibility of employment could lead to a better psychological state in their last academic year [9].

The higher GHQ-28 scores that nursing students obtained in the different academic years of the nursing programs indicate that, during some academic years, nursing students are prone to somatic symptoms, anxiety and insomnia, social dysfunction, and depression. The presence of higher scores in some dimensions of the GHQ-28 when comparing different academic years and universities reveals that the second academic year is probably the most challenging. We observed that students in the second academic year scored higher, which could indicate a higher risk of suffering from a mental health disorder during this specific year of the degree. The fact that most nursing students proceeded directly to the bachelor’s degree could explain this tendency. Although we observed lower scores in fourth-year students as well, we cannot confirm that this tendency is caused by the academic curricula as this study has a cross-sectional design and compares independent groups. Those students whose academic files were transferred were mainly in their 2nd or 3rd year, which is consistent with the fact that there is a minimal requirement of credits to apply [17]. Therefore, it seems that being at UFRO carries a greater risk of scoring above 23, which means a greater risk of psychiatric pathology. However, as mentioned above, these results may not be generalizable given the nature of the study. It is worth noting that the variations in the GHQ-28 scores found in UFRO students may be due to intercultural differences when evaluating common psychiatric disorders [32].

Concerning the perception of the stress of the student experience according to the different academic methods, all but one (oral presentations) were reported as highly stressful, which agrees with the existing literature [13,33]. In the literature review by Mccarthy et al., they found that clinical stressors were equally relevant to academic stressors, which included the academic environment, examinations, and assignments. The correlation findings in our study showed that the low level of well-being was related to stress due to exams, seminars, clinical practices, group projects, and PBL methodologies. It seems that those methods in which students are required to play an active role in the classroom are sources of greater stress than those where they have a passive role. Despite the learning benefits that may be observed over time [5,34], students are unaware of these new methods until their first year of university, thus they are a critical source of stress for them. Furthermore, the PBL methodology has recently been integrated into the nursing teaching programs, and its frequent use throughout the nursing curricula allows students to acquire a certain degree of expertise [35]. Consequently, the regression model showed that the stress generated by seminars, PBL, group projects, and exams had a direct impact on nursing students’ risk of suffering from a mental health illness, thus generating a low to moderate degree of perception of psychological distress.

The results obtained allowed us to identify some of the factors that could be associated with the psychological well-being of nursing students. Based on the analysis of the results, we formulated the hypothesis that if students are more advanced in the nursing degree curricula, their psychological well-being will progressively improve. Moreover, we propose a longitudinal study of one cohort of students throughout the nursing degree and reassess the factors identified in this study and their association with the psychological well-being of the cohort.

### Limitations

The cross-sectional design chosen for this study does not allow us to conclude a predictive relationship of the studied variables or to analyze other variables that may have affected mental health in nursing students (sense of coherence, emotional exhaustion, self-esteem, or personality). Longitudinal data are necessary to confirm changes in the well-being of the participants, especially those associated with each stage of the nursing program. Another limitation of this study was that it did not analyze specific variables or constructs related to well-being (such as the development of personal and social abilities) and attitudes (such as personal resilience); or mediating variables (such as the dispositional optimism associated with stress reduction). It is worth mentioning that the questionnaires used were self-reported, since participants may feel vulnerable providing researchers access to their psychological status. This could have resulted in some biased answers [36]. Nonetheless, self-reported measures provide accurate information and offer a realistic approximation of the first-hand experiences of each individual.

## 5. Conclusions

Our results suggest that academic stress is associated with the GHQ-28 scores. Despite the high level of stress due to the teaching methodologies in all courses, being a student in the last year of the degree indicates better psychological well-being when compared to students in their first year.

These findings could indicate that formal training and education in nursing, added to other personal factors, could help students to develop protective mechanisms against possible mental disorders. Educators face the challenge of balancing proven learning methodologies and their role in their students’ mental health. Finally, further longitudinal studies are needed to determine the future impact of these outcomes.

## Figures and Tables

**Table 1:** Sociodemographic and academic characteristics.

	Total	1st Year *N* (%)	2nd Year *N* (%)	3rd Year *N* (%)	4th Year *N* (%)	*p*-Value
Age—Median (IQR)	21.0 (3.0)	20.0 (3.0)	21.0 (3.0)	22.0 (2.0)	23.0 (3.0)	<0.001
Age by range						
≤20	488 (36.4)	274 (65.7)	153 (38.2)	59 (16.3)	2(1.2)	<0.001
21–24	640 (47.7)	93 (22.3)	184 (45.9)	248 (68.5)	115 (71.4)	
25–28	126 (9.4)	21 (5.0)	38 (9.5)	34 (9.4)	33 (20.5)	
29–32	31 (2.3)	9 (2.2)	8 (2.0)	8 (2.2)	6 (3.7)	
33–36	29 (2.2)	11 (2.6)	8 (2.0)	7 (1.9)	3 (1.9)	
>37	27 (2.0)	9 (2.2)	10 (2.5)	6 (1.7)	2 (1.2)	
Gender						
Female	1108 (83.7)	347 (84.8)	333 (83.9)	294 (82.6)	134 (83.2)	0.861
Male	215 (16.3)	62 (15.2)	64 (16.1)	62 (17.4)	27 (16.8)	
Marital status						
Single	1173 (86.8)	368 (87.8)	356 (88.1)	303 (83.0)	146 (89.6)	0.089
Married	34 (2.5)	11 (2.6)	8 (2.0)	10 (2.7)	5 (3.1)	
Steady partner	128 (9.5)	34 (8.1)	33 (8.2)	49 (13.4)	12 (7.4)	
Divorced	7 (0.5)	1 (0.2)	5 (1.2)	1 (0.3)	0 (0.0)	
Access route to university studies						
University access test	1015 (74.9)	339 (80.0)	282 (70.1)	281 (76.8)	113 (69.3)	<0.001
Up to 25 years test *	41 (3.0)	16 (3.8)	12 (3.0)	11 (3.0)	2 (1.2)	
Up to 45 years test **	7 (0.5)	4 (0.9)	2 (0.5)	0 (0.0)	1 (0.6)	
Vocational training	231 (17.0)	43 (10.1)	81 (20.1)	65 (17.8)	42 (25.8)	
Other university degree	50 (3.7)	20 (4.7)	17 (4.2)	8 (2.2)	5 (3.1)	
University						
University of Lleida	319 (23.5)	91 (21.5)	92 (22.8)	83 (22.7)	53 (32.3)	<0.001
University of Girona	387 (28.5)	123 (29.0)	98 (24.3)	113 (30.9)	53 (32.3)	
University Rovira I Virgili (Tarragona)	491 (36.2)	167 (39.4)	188 (46.5)	96 (26.2)	40 (24.4)	
Universidad de la Frontera (Chile)	161 (11.9)	43 (10.1)	26 (6.4)	74 (20.2)	18 (11.0)	
Academic characteristics						
Transfer of academic file						
Yes	87 (6.4)	11 (2.6)	32 (8.0)	31 (8.5)	13 (7.9)	0.008
No	1261 (93.5)	410 (97.4)	367 (91.8)	333 (91.5)	151 (92.1)	
Clinical practice experience						
Yes	1081 (79.8)	160 (37.8)	396 (98.5)	362 (98.9)	163 (100)	<0.001
No	269 (19.9)	261 (61.7)	5 (1.2)	3 (0.8)	0 (0.0)	
Financial burden						
Working						
Yes	429 (31.7)	121 (28.6)	119 (29.6)	127 (34.8)	62 (38.0)	0.040
No	922 (68.1)	302 (71.4)	283 (70.4)	283 (70.4)	100 (61.3)	
Weekly working hours—Median (IQR)	15.0 (16.0)	16.0 (17.0)	15.0 (17.0)	15.0 (14.0)	20.0 (10.0)	0.397
Funding system						
Own	244 (18.0)	70 (16.6)	73 (18.1)	64 (17.5)	37 (22.6)	0.020
Family	842 (62.2)	280 (66.5)	260 (64.5)	217 (59.5)	85 (51.8)	
Both	267 (19.7)	71 (16.9)	70 (17.4)	84 (23.0)	42 (25.6)	
Scholarship awarded						
Yes	735 (54.2)	223 (52.7)	218 (54.0)	204 (55.7)	90 (54.9)	794
No	621 (45.8)	200 (47.3)	185 (45.8)	162 (44.3)	74 (45.1)	
Family responsibilities						
Children						
0	1243 (95.3)	382 (95.3)	372 (95.6)	338 (95.2)	151 (95.0)	0.309
1	42 (3.2)	12 (3.0)	9 (2.3)	13 (3.7)	8 (5.0)	
2	17 (1.3)	7 (1.7)	6 (1.5)	4 (1.1)	0 (0.0)	
3	2 (0.2)	0 (0.0)	2 (0.5)	0 (0.0)	0 (0.0)	
Dependents						
Yes	21 (1.6)	5 (1.2)	5 (1.3)	7 (1.9)	4 (2.5)	0.608
No	1312 (98.4)	411 (98.8)	390 (98.7)	357 (98.1)	154 (97.5)	

Quantitative variables, such as age and weekly working hours, are expressed with median (IQR), and the *p*-value is obtained using the Kruskal–Wallis test. Qualitative variables are presented with the number of cases (percentage), and the *p*-value is calculated through the Chi-squared test. */**: Up to 25 * and 45 ** years test: according to Spanish law, people over twenty-five years of age or forty-five of years who pass the entrance test established in the royal decree 412/2014, June, 6th [23].

**Table 2:** General Health Questionnaire scores (dimensions and total score) by course.

	Total	1st Year	2nd Year	3rd Year	4th Year	*p*-Value
GHQ Somatic symptoms (A)	5.0 (3.0)	5.0 (3.0)	5.0 (3.0)	5.0 (3.0)	4.0 (4.0)	0.056
GHQ Anxiety and Insomnia (B)	6.0 (3.0)	6.0 (3.0)	6.0 (3.0)	6.0 (3.0)	5.0 (4.0)	0.170
GHQ Social Dysfunction (C)	6.0 (2.0)	6.0 (2.0)	6.5 (2.0)	6.0 (2.0)	6.0 (2.0)	0.007
GHQ Depression (D)	1.0 (2.0)	1.0 (2.0)	1.0 (2.0)	1.0 (2.0)	0.0 (1.0)	0.001
Total GHQ-28	23.0 (15.0)	24.0 (14.0)	24.0 (14.0)	23.0 (15.0)	19.0 (15.0)	<0.001

Data are presented with median (IQR), and the *p*-value is obtained using the Kruskal–Wallis test. GHQ = General Health Questionnaire.

**Table 3:** Stress levels by different learning methodologies per academic year.

	Total	1st Year	2nd Year	3rd Year	4th Year	*p*-Value
Lecture	2.0 (1.0)	2.0 (1.0)	2.0 (1.0)	2.0 (1.0)	2.0 (1.0)	<0.001
Seminar	2.0 (1.0)	2.0 (1.0)	2.0 (1.0)	2.0 (1.0)	2.0 (1.0)	<0.001
Problem-based learning (PBL)	3.0 (1.0)	3.0 (1.0)	3.0 (2.0)	3.0 (1.0)	3.0 (1.0)	<0.001
Simulation laboratory	3.0 (1.0)	2.0 (1.0)	3.0 (1.0)	3.0 (2.0)	3.0 (2.0)	<0.001
Clinical practice	3.0 (1.0)	2.0 (1.0)	3.0 (1.0)	3.0 (1.0)	3.0 (1.0)	<0.001
Group Project	3.0 (1.0)	3.0 (1.0)	3.0 (1.0)	3.0 (1.0)	2.0 (1.0)	<0.001
Exams	4.0 (1.0)	4.0 (1.0)	4.0 (1.0)	4.0 (1.0)	4.0 (1.0)	<0.001
Written work	3.0 (1.0)	3.0 (1.0)	3.0 (1.0)	3.0 (1.0)	3.0 (1.0)	0.003
Oral presentation	4.0 (1.0)	4.0 (1.0)	4.0 (1.0)	3.0 (1.0)	3.0 (1.0)	0.417

Data are presented as median (IQR). Levels of response: 1: None, 2: Low, 3: Moderate; 4: High. *p*-value is calculated through the Kruskal–Wallis test.

**Table 4:** Logistic regression model to evaluate the effects of sociodemographic and academic characteristics, together with the stress caused by different types of training activities on having a total GHQ-28 score higher than 23.

Variables	Odds Ratio (OR)	Lower 95%CI OR	Upper 95% CI OR	*p*-Value
Constant	0.017			<0.001
Gender				
Female	1.0			
Male	0.655	0.456	0.940	0.022
University				
UdL	1.0			
URV	1.260	0.868	1.830	0.224
UdG	1.398	0.944	2.069	0.094
UFRO	3.008	1.694	5.338	<0.001
Seminar-related stress	1.426	1.163	1.748	0.001
PBL-related stress	1.265	1.066	1.501	0.007
Group project-related stress	1.246	1.068	1.454	0.005
Exam-related stress	1.591	1.274	1.986	<0.001

Abbreviations: UdL: Lleida University; URV: Universitat Rovira i Virgili; UdG: Universitat de Girona; UFRO: Universidad de la Frontera.

## Data Availability

The data presented in this study are available on request from the corresponding author. The data are not publicly available as there is new data and it has not yet been published.

## Supplementary Materials

The following are available online at https://www.mdpi.com/1660-4601/18/6/3020/s1, Table S1: STROBE Statement—Checklist of items that should be included in reports of cross-sectional studies.
